# Molecular Insights into the Temperature-Dependent Binding and Conformational Dynamics of Noraucuparin with Bovine Serum Albumin: A Microsecond-Scale MD Simulation Study

**DOI:** 10.3390/ph18071048

**Published:** 2025-07-17

**Authors:** Erick Bahena-Culhuac, Martiniano Bello

**Affiliations:** 1 Laboratorio de Diseño y Desarrollo de Nuevos Fármacos e Innovación Biotecnológica, Sección de Estudios de Posgrado e Investigación, Escuela Superior de Medicina, Instituto Politécnico Nacional, Plan de San Luis y Salvador Diaz Mirón s/n, Casco de Santo Tomás, Miguel Hidalgo, Ciudad de México 11340, Mexico; erickb_2000@hotmail.com; 2 Faculty of Biology and Medicine, University of Lausanne, 1015 Lausanne, Switzerland

**Keywords:** noraucuparin, bovine serum albumin (BSA), molecular dynamics simulations, MMGBSA, protein–ligand binding

## Abstract

**Background/Objectives**: Understanding the molecular interactions between small bioactive compounds and serum albumins is essential for drug development and pharmacokinetics. Noraucuparin, a biphenyl-type phytoalexin with promising pharmacological properties, has shown a strong binding affinity to bovine serum albumin (BSA), a model protein for drug transport. This study aims to elucidate the structural and energetic characteristics of the noraucuparin–BSA complex under physiological and slightly elevated temperatures. **Methods**: Microsecond-scale molecular dynamics (MD) simulations and Molecular Mechanics Generalized Born Surface Area (MMGBSA)-binding-free energy calculations were performed to investigate the interaction between noraucuparin and BSA at 298 K and 310 K. Conformational flexibility and per-residue energy decomposition analyses were conducted, along with interaction network mapping to assess ligand-induced rearrangements. **Results**: Noraucuparin preferentially binds to site II of BSA, near the ibuprofen-binding pocket, with stabilization driven by hydrogen bonding and hydrophobic interactions. Binding at 298 K notably increased the structural mobility of BSA, affecting its global conformational dynamics. Key residues, such as Trp213, Arg217, and Leu237, contributed significantly to complex stability, and the ligand induced localized rearrangements in the protein’s intramolecular interaction network. **Conclusions**: These findings offer insights into the dynamic behavior of the noraucuparin–BSA complex and enhance the understanding of serum albumin–ligand interactions, with potential implications for drug delivery systems.

## 1. Introduction

Bovine serum albumin (BSA) is one of the most abundant and well-characterized water-soluble transport proteins. BSA functions as an in vivo carrier [1], and its active binding sites have been extensively studied [2]. Its primary structure consists of nine loops connected by 17 disulfide bonds, organized into three domains (I, II, III), each further subdivided into two subdomains (A and B). BSA is a well-studied protein composed of 583 amino acid residues, with a molecular weight of 66.4 kDa, an isoelectric point (pI) of 4.9, and a spherical, heart-like shape [3].

BSA contains two tryptophan residues: Trp134, located on the surface of the first domain, and Trp213, situated in a hydrophobic binding pocket of the second domain. The protein exhibits a high binding affinity for a wide range of drugs and metabolites [4], making it one of the most extensively investigated proteins. Its surface is enriched with charged amino acids, which enhance its interaction with various compounds.

Several X-ray crystal structures of BSA have been resolved, providing detailed insights into its ligand-binding sites. For instance, the PDB structure 4F5S features BSA complexed with warfarin at Site I, while 6QS9 shows a ketoprofen bound at the same site [5,6]. These structures have been instrumental in differentiating between the two principal drug-binding sites originally characterized by Sudlow et al. (1975): Site I, located in subdomain IIA of Domain II, and Site II, located in subdomain IIIA of Domain III [7]. BSA’s three homologous domains (I, II, and III), each subdivided into A and B subdomains, form the structural framework for these binding pockets. Site I and Site II are defined by their distinct ligand-binding preferences and interaction profiles, rather than by entire domains. The identification of specific residues within these subdomains provides a deeper understanding of ligand selectivity and binding affinity.

**Figure 1:**
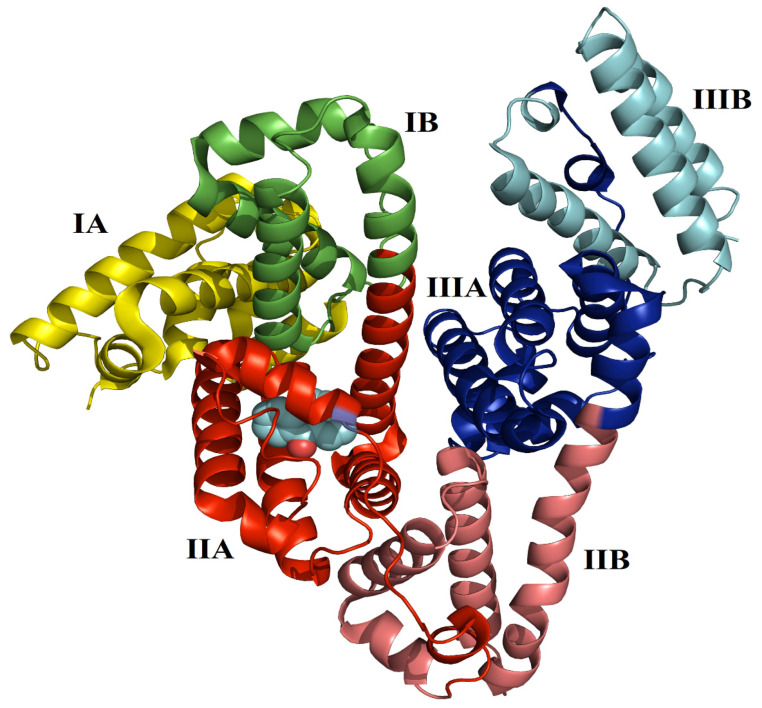
Architecture of the structure of BSA site II. BSA contains three homologous α-helical domains (I, II, III), which contain two subdomains (A and B). This figure was constructed using PyMol v0.99 software.

A high likelihood has been observed for fatty acids (FAs) to bind at subdomain IIA of BSA through interactions involving hydrophobic forces, van der Waals, and electrostatic interactions [8]. As depicted in Figure 1, subdomain IIA forms a hydrophobic pocket composed of Leu237, Tyr149, Arg256, Glu152, Ser191, Tyr156, Glu291, Ala290, Arg198, Arg217, and His241. Although it has been reported that Arg256 also plays an important role in FA stabilization through hydrogen bonding [9], the residues forming the hydrophobic pocket further stabilize the complex [10].

These sites facilitate the binding and transport of relatively insoluble endogenous and exogenous compounds, including various drugs. These substances can exist either freely in the plasma’s aqueous phase or as complexes bound to the protein. BSA plays key roles in the body, including molecule transport, regulation of tissue and organ functions, catalysis of biochemical reactions, and modulation of dissolved gases and foreign particles [11]. Numerous studies have investigated the binding interactions between small molecules and BSA, highlighting its importance in biological and pharmaceutical contexts [12,13,14,15].

**Scheme 1:**
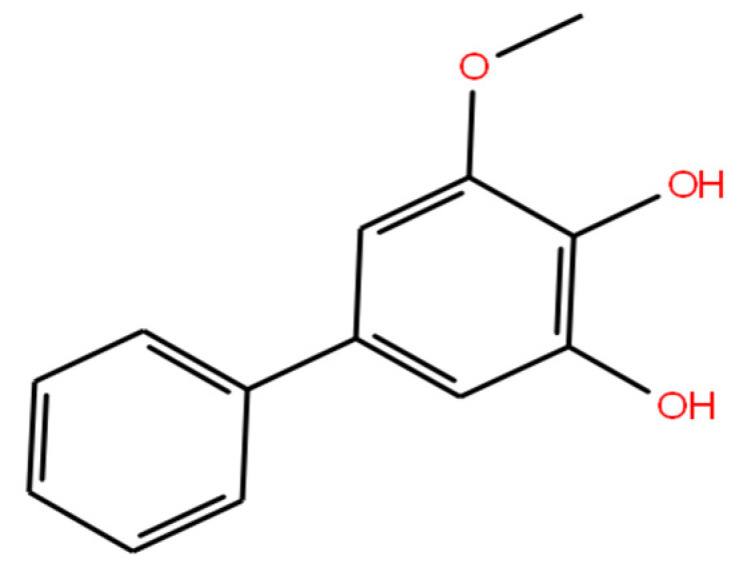
Chemical structure of noraucuparin, a biphenyl-type phytoalexin bearing two hydroxyl groups and one methoxy group on the substituted aromatic ring.

Noraucuparin, a biphenyl-type phytoalexin (Scheme 1) derived from plants, is typically isolated from the leaves of Sorbus pohuashanensis. It has recently gained significant interest due to its broad pharmacological benefits and therapeutic promise [16,17,18]. This compound displays a range of biological activities, including antioxidant, antimicrobial, anticancer, and anti-inflammatory effects, making it a strong candidate for drug development and therapeutic applications [19,20,21,22].

Noraucuparin contains aromatic biphenyl rings that facilitate π–π stacking interactions with biomolecules, notably with Trp213 of BSA, as well as with plant biosynthetic enzymes, such as O-methyltransferase and cinnamate-CoA ligase, which participate in biphenyl phytoalexin metabolism [23,24].

Moreover, its chemical structure contains hydroxyl groups and other functional moieties, which contribute to its physicochemical characteristics and enhance its pharmacological properties. Antibacterial activity of Noraucuparin has been observed in recent studies [25], although the precise molecular mechanism remains to be fully elucidated.

Recent experimental and computational studies employing fluorescence spectroscopy, circular dichroism (CD), isothermal titration calorimetry (ITC), and molecular docking have provided insight into the thermodynamic parameters of binding and conformational changes in BSA upon interaction with noraucuparin at 298 and 310 K [26]. Experimental methods revealed a favorable binding-free energy (∆G) between Noraucuparin and BSA in a 1:1 stoichiometric ratio, accompanied by changes in BSA’s secondary structure upon molecular recognition, suggesting a disruption of the hydrogen bond network within BSA. Meanwhile, docking simulations identified that Noraucuparin binds to Site II on BSA, near the ibuprofen-binding site, with the interaction stabilized by a hydrogen bond between Noraucuparin’s hydroxyl group and the Ser453 residue of BSA. Additionally, experimental studies at 298 and 310 K demonstrated an increase in affinity with rising temperatures.

Computational approaches, such as molecular docking, MD simulations, and quantum mechanical analyses, are widely employed to predict ligand–protein interactions and pharmacokinetic behavior. Recent studies have highlighted the value of these methods in identifying bioactive compounds for neurological and systemic diseases, including the theoretical evaluation of 1,2-dihydroquinoline derivatives as potential treatments for multiple sclerosis [27].

Long-timescale MD simulations have proven to be highly valuable in capturing relevant conformational changes, binding events, and the dynamic behavior of biomolecular assemblies that are often inaccessible with short simulations [28,29,30,31,32,33]. These extended simulations allow for a more accurate exploration of the protein–ligand energy landscape, including metastable states and slow collective motions associated with binding and allosteric regulation.

Accordingly, in this study, and building on the experimental and theoretical results reported by Dai et al. (2025) [26], we employed blind molecular docking followed by 1 μs, triplicate, independent MD simulations to investigate the interaction between noraucuparin and BSA. Our aim was to characterize the structural and energetic features of this interaction under both physiological and elevated temperature conditions.

## 2. Results

### 2.1. Stability of Simulated Systems

The RMSD (Root Mean Square Deviation), radius of gyration (Rg), and surface area values represent the structural deviations of the protein over time, providing insights into system stability. Each panel (A–D) in Figures S1–S3 corresponds to a different system, while the black, blue, and red lines within each panel represent three independent MD simulations (replicates) of the same system. In all cases, RMSD, Rg, and surface area increase rapidly during the initial phase (~0–0.5 µs) due to equilibration, after which they stabilize, indicating that each system has reached equilibrium. The RMSD, Rg, and surface area values for each system are relatively consistent across the three replicates, suggesting that the results are reproducible and not merely due to random fluctuations. Some variability between replicates is observed, particularly in BSAfree-298K (A) and BSAbound-298K (C), where one replicate exhibits slightly higher deviations. This suggests that, while the overall structural behavior of each system remains consistent, local conformational differences may arise between independent runs.

It is important to note that the RMSD values discussed here were calculated using the Cα atoms of the protein only, even in the bound systems. This allows for a direct comparison of protein backbone stability across all conditions, independent of the ligand. The slightly higher RMSD fluctuations observed in BSAfree-310K and BSAbound-298K are not indicative of structural instability, but rather suggest an enhanced conformational flexibility in response to the temperature and ligand binding. This interpretation is further supported by the Root Mean Square Fluctuation (RMSF) analysis and the broader phase space projections observed in the BSAbound-298K system. In particular, ligand-induced flexibility at 298 K appears to affect specific loop regions, contributing to the observed increase in RMSD without compromising the overall structural integrity. Thus, the RMSD data reflect meaningful dynamic adaptations of BSA under different conditions, consistent with its biological role as a flexible carrier protein.

### 2.2. RMSF Analysis

**Figure 2:**
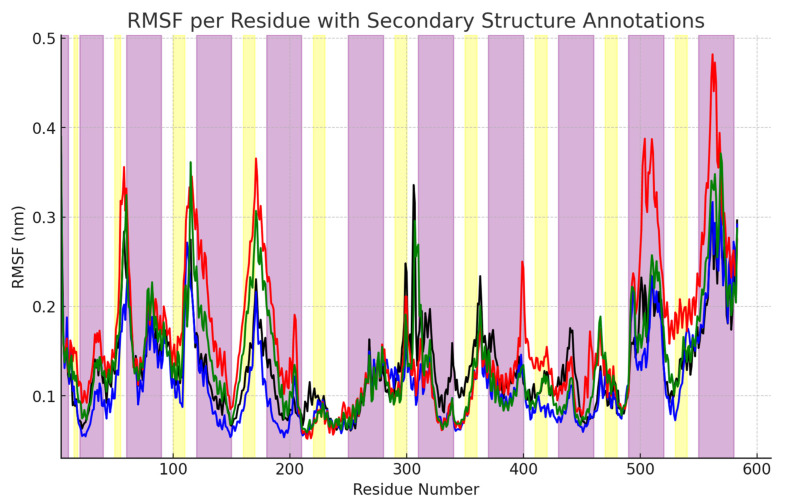
RMSF analysis of free and bound BSA at 298 and 310 K. The RMSF values (in nm) were calculated for the Cα atoms of BSA in four systems: BSA_free-298K_ (black), BSA_free-310K_ (blue), BSA_bound-298K_ (red), and BSA_bound-310K_ (green). Secondary structure elements are annotated with purple shading for α-helices and yellow shading for β-sheets.

As part of our objective to understand how temperature influences the conformational flexibility of BSA upon ligand binding, we analyzed the RMSF graph and statistical data (Figure 2 different lines). Based on this analysis, the most flexible regions are as follows: Residues ~1–10 (N-terminal region): This region exhibits high RMSF values across all systems due to its lack of a secondary structure, making it inherently flexible. Residues ~200–230 (Loop between H12 and H13): This flexible loop shows a noticeable increase in RMSF, especially in BSAbound-298K (red). Residues ~310–340 (Loop between H17 and H19): Another highly fluctuating region, suggesting structural flexibility that may be functionally relevant. Residues ~500–550 (Near H29 and H30): This loop exhibits significant fluctuations, particularly in BSAbound-298K and BSAbound-310K, indicating that ligand binding increases the flexibility in this region. These regions correspond to loops and unstructured segments, which naturally exhibit greater mobility compared to more stable α-helices and β-sheets [34].

Analyzing the mean RMSF values, we can determine which system exhibited the most flexibility: BSAbound-298K (red) had the highest RMSF values, with a mean RMSF of 0.161 nm, indicating the greatest overall mobility. This suggests that ligand binding at 298 K increases the protein flexibility, especially in loop regions. BSAbound-310K (green) also showed increased flexibility, but slightly lower than at 298 K, with a mean RMSF of 0.138 nm. This indicates that higher temperatures stabilize some of the fluctuations induced by ligand binding. BSAfree-298K (black) and BSAfree-310K (blue) exhibited the lowest RMSF values, with 0.133 nm and 0.117 nm, respectively. This suggests that the unbound protein is more rigid, and temperature alone does not significantly increase the flexibility in the absence of a ligand.

### 2.3. Correlation Between RMSF and Ligand Binding in the Binding Site

**Figure 3:**
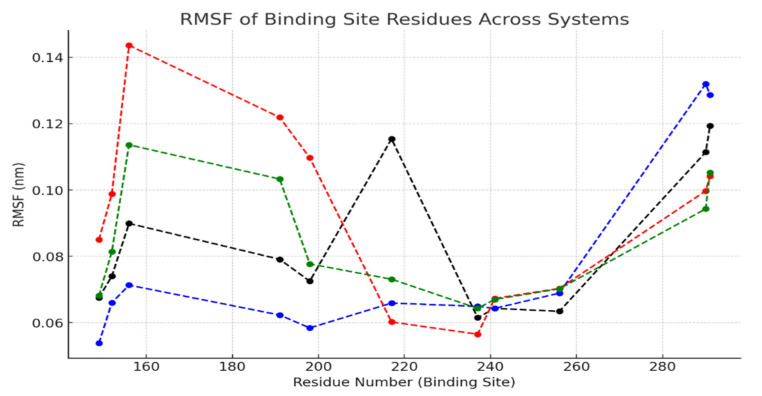
RMSF of binding-site residues across the different systems: The black, blue, red, and green lines represent BSA_free-298K_, BSA_free-310K_, BSA_bound-298K_, and BSA_bound-310K_, respectively. The dots represent specific residues within the binding-site region that are involved in or proximal to ligand interactions.

Based on the RMSF values of the binding-site residues and their comparison across the four systems (Figure 3), we can observe the following trends: (1) Increased Flexibility in the Bound Systems: The BSAbound-298K (red) and BSAbound-310K (green) systems show higher RMSF values in multiple binding-site residues compared to the BSAfree systems. This suggests that ligand binding induces conformational flexibility within the binding site, potentially due to ligand-induced adaptations in the hydrophobic pocket, as previously observed for other small molecules binding to BSA [9]. (2) Residues Most Affected by Ligand Binding from the RMSF data: The following residues exhibited significant changes in mobility upon ligand binding: Leu237, Arg256, and Arg217 showed increased RMSF in the bound systems. Tyr149 and Tyr156 also displayed moderate fluctuations, which may indicate conformational adjustments of these residues to accommodate the ligand (Figure 3). (3) Effect of Temperature: The BSA–ligand complex at 310 K (green) showed lower flexibility in the binding site compared to 298 K, indicating that a higher temperature may help stabilize certain movements within the protein. In contrast, the unbound BSA systems (black and blue) consistently exhibited lower flexibility overall, which supports the idea that the binding site stays more rigid when no ligand is present.

### 2.4. Phase Space Projection of Free and Bound BSA at Different Temperatures

**Figure 4:**
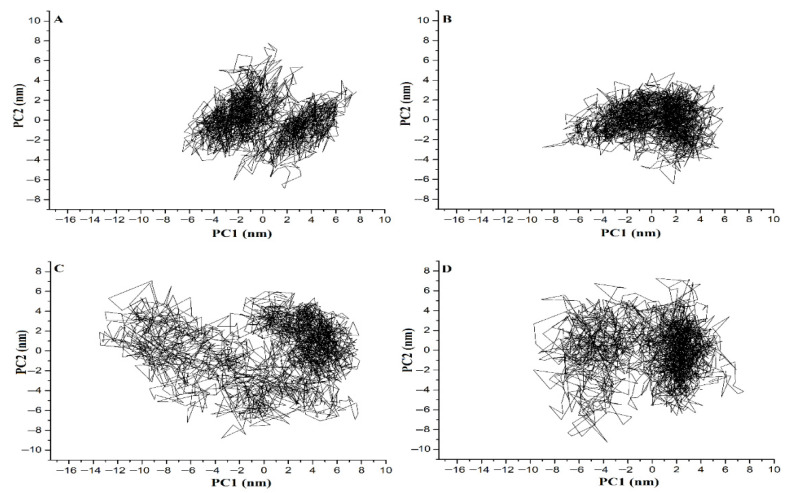
Projection of free and bound BSA at 298 and 310 K in phase space. Projection of motion in phase space for (**A**) BSA_free-298K_, (**B**) BSA_free-310K_, (**C**) BSA_bound-298K_, and (**D**) BSA_bound-310K_. Graphs correspond to average values of three different MD simulations for each system.

To further investigate the effects of ligand binding and temperature on BSA’s conformational dynamics, we analyzed the phase space projection of the free and bound systems. By analyzing the phase space projections at different temperatures, we can assess how ligand binding influences the conformational landscape of BSA (Figure 4). Comparing the free and bound states at 298 K shows that BSAbound-298K (Figure 4C) has a broader phase space projection than BSAfree-298K (Figure 4A), suggesting that ligand binding increases flexibility at 298 K. In contrast, free BSA at 298 K (Figure 4A) explores a more limited conformational space, possibly stabilizing around fewer dominant conformations. This implies that ligand binding at 298 K does not rigidify the BSA but instead enhances its conformational exploration. This effect could be attributed to allosteric interactions or ligand-induced conformational shifts that disrupt stabilizing intramolecular interactions, as described in the context of signal transduction and protein dynamics [35].

The comparison of BSAfree-310K (B) vs. BSAbound-310K (D) shows that BSAfree-310K (B) shows a narrower projection than BSAbound-310K (D), indicating that ligand binding also increases flexibility at 310 K. Similarly to the trend observed at 298 K, BSAfree-310K (B) displays a narrower projection, suggesting that without the ligand, the protein explores fewer conformational states. This finding suggests that, at both temperatures (298 K and 310 K), ligand binding destabilizes certain intramolecular interactions, leading to a wider conformational landscape.

### 2.5. MD Simulations of BSA–Ligand Interactions

The investigation into the interactions between BSA and Noraucuparin at 298 K and 310 K revealed notable differences in the number of stabilizing amino acid residues involved. At 298 K, the complex was stabilized by interactions with 11 residues (Arg198, Trp213, Arg217, Leu218, Leu237, Val240, His241, Arg256, Leu259, Ile289, and Ala290), while at 310 K, the interaction was mediated by 9 residues (Trp213, Arg217, Leu218, Leu237, His241, Arg256, Leu259, Ile289, and Ala290) (Figure 2). A key observation is that Trp213, Arg217, Leu218, Leu237, His241, Arg256, Leu259, Ile289, and Ala290 were consistently involved in stabilizing the complex at both temperatures, suggesting that these residues play a fundamental role in Noraucuparin binding.

#### Hydrogen Bonding and Structural Insights

**Figure 5:**
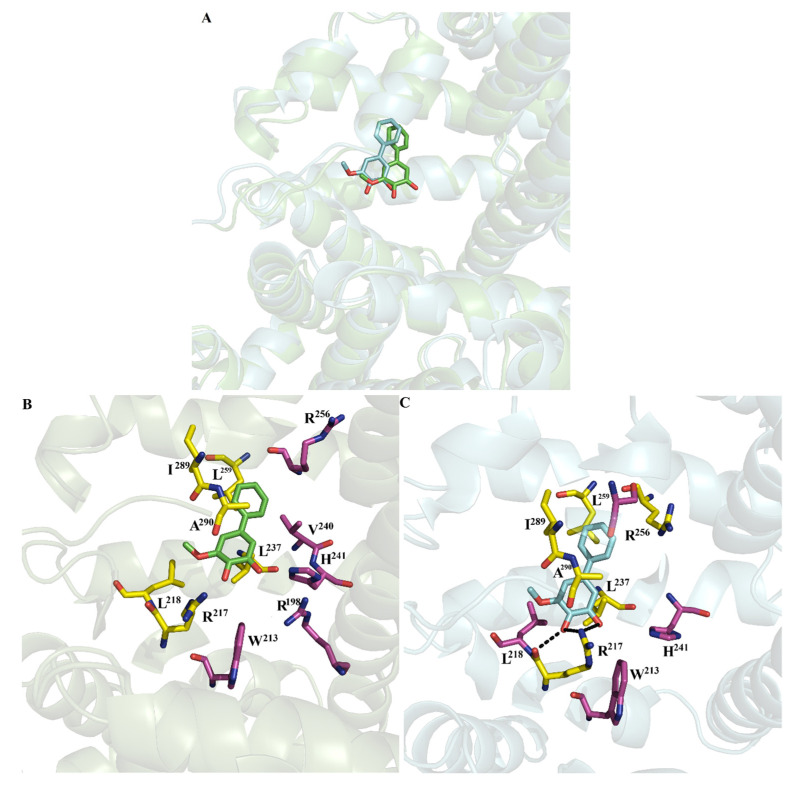
Receptor–ligand interactions of the complexes between the BSA and ligand at 298 and 310 K. (**A**) Structural overlap of the BSAbound-298K and BSAbound-310K systems, (**B**) BSAbound-298K, and (**C**) BSAbound-310K. Panel (**A**) provides a general overview of the BSA–Noraucuparin complex, illustrating how the ligand is positioned within its binding site. The conformations were obtained via a cluster analysis over the last 200 ns of simulations.

Hydrogen bonding was observed at 310 K, contributing to the stability of the complex. The catechol ring of Noraucuparin forms hydrogen bonds through its two hydroxyl groups (-OH) with atoms from the side chain and specifically the guanidinium group of Arg217 (Figure 5B,C). These interactions likely contribute to the specificity and stability of the ligand–protein binding.

The hydrogen-bonding analysis based on trajectory-derived occupancy data confirms that Arg217 forms a hydrogen bond with Noraucuparin at both 298 K and 310 K. This bond is significantly more stable at 310 K, persisting for approximately 33% of the simulation compared to only 10% at 298 K (see Supplementary Figure S4).

Additionally, a comparative structural analysis (Figure 5B,C) highlights the hydrophobic pocket formed by Leu218, Leu237, Ile289, and Ala290, which may further enhance ligand binding through van der Waals interactions. The role of Trp213 is particularly interesting, as it is positioned in proximity to the ligand and could contribute to binding through π-π stacking interactions.

The differences in binding interactions at 298 K and 310 K suggest a potential entropic contribution to ligand affinity. At higher temperatures, the binding mode appears more refined, relying on a selective subset of stabilizing residues while preserving strong hydrogen bonding. This effect has been associated with entropic optimization in ligand–protein complexes [36].

### 2.6. Binding-Free Energy Calculations Using MMGBSA

**Figure 6:**
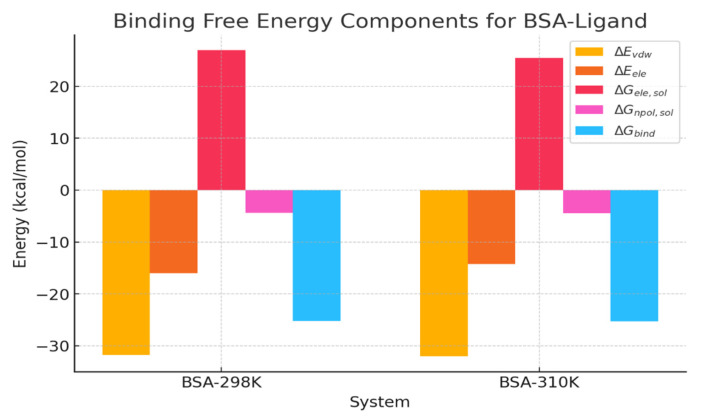
Binding-free energy components for BSA–ligand at 298 K and 310 K during the last 0.2 microsecond from one-microsecond triplicate MD simulations. The energy components include van der Waals interaction energy (ΔEvdw), electrostatic interaction energy (ΔEele), solvation-free energy (polar component, ΔGele,sol, and non-polar component, ΔGnpol,sol), and the total binding-free energy (ΔGbind). Energy values are expressed in kcal/mol.

We calculated the binding-free energy (ΔGbind) using the MMGBSA approach. As shown in Figure 6, the binding energies at both temperatures are consistently negative, which indicates that the ligand binds stably to the protein. The values are −25.26 ± 3.2 kcal/mol at 298 K and −25.30 ± 1.4 kcal/mol at 310 K, suggesting that the difference in temperature has minimal impact on the overall binding strength.

However, the electrostatic interactions (ΔEele) and polar solvation energies (ΔGele,sol) show higher variability at 310 K, which might indicate slight conformational fluctuations at higher temperatures. Van der Waals and hydrophobic contributions, expressed by ΔEvdw values, remain stable (~−32 kcal/mol at both temperatures), suggesting that hydrophobic interactions dominate ligand binding. The non-polar solvation energy (ΔGnpol,sol) is consistently negative (~−4.4 kcal/mol), reinforcing the role of hydrophobic stabilization in ligand binding, as commonly observed in protein–ligand systems [37].

Electrostatic interactions and solvation effects (ΔEele) show higher variation at 310 K, possibly due to increased molecular motion and solvent effects. Polar solvation (ΔGele,sol) is slightly lower at 310 K, which might indicate differences in water interactions with the binding site.

Overall, we can summarize that the binding affinity remains strong and is not significantly affected by temperature changes. Hydrophobic interactions (van der Waals and non-polar solvation) play a crucial role in stabilizing the ligand within the binding pocket. Electrostatic interactions and solvation effects show some temperature-dependent variations, but they do not drastically alter the binding-free energy.

To further explore the temporal evolution of binding affinity, ΔG values were also computed over four sequential 0.2 μs intervals spanning the entire 1 µs trajectory (Figure S6). This extended analysis confirmed the stability of the complex across all intervals, with a slightly more favorable average ΔGbind at 298 K (−26.11 ± 0.56 kcal/mol) than at 310 K (−25.38 ± 0.41 kcal/mol). These results complement the values from the final 0.2 μs and highlight the consistency of ligand binding throughout the simulation.

### 2.7. Per-Residue Free Energy Decomposition for Complexes Between BSA and Noraucuparin at 298 K and 310 K

**Figure 7:**
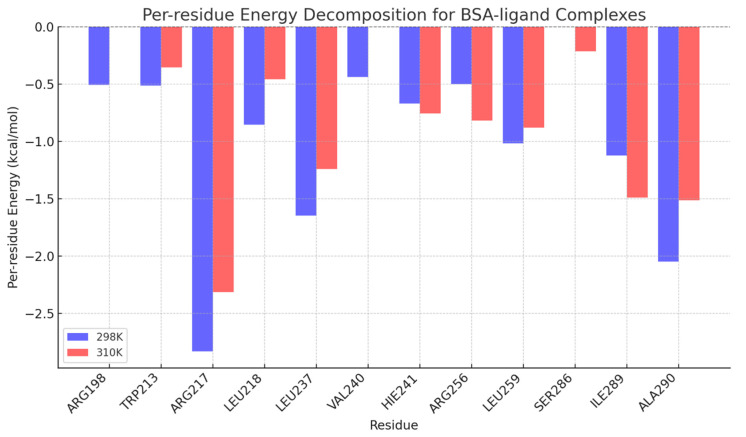
Per-residue energy decomposition for BSA–ligand complexes at 298 K and 310 K during the last 0.2 microseconds from one-microsecond triplicate MD simulations. The energy values (kcal/mol) represent the contribution of each residue to the binding-free energy. Blue bars correspond to 298 K, while red bars represent 310 K. Negative values indicate stabilizing interactions.

Figure 7 shows that most residues exhibit less favorable binding energies at 310 K compared to 298 K. This suggests that an increase in temperature slightly weakens residue–ligand interactions, likely due to increased molecular motion and entropy. For Trp213, the energy contribution decreases from −0.51 kcal/mol at 298 K to −0.35 kcal/mol at 310 K, indicating a weakening of its interaction with the ligand. As Trp213 is known to play a role in hydrophobic interactions, this may suggest reduced van der Waals interactions due to increased thermal fluctuations.

Although Arg217 contributes favorably at both temperatures (−2.83 kcal/mol at 298 K to −2.31 kcal/mol at 310 K), the slight decrease in energy contribution at 310 K suggests that while hydrogen bonding is present at both temperatures (Figure 5B,C), at 298 K, Arg217 contributes more through electrostatic interactions, which are more transient and influenced by solvent effects. In contrast, at 310 K, the higher prevalence of hydrogen bonding indicates a more defined and structured binding mode, favoring a specific ligand recognition pattern over broader electrostatic stabilization.

For Leu218 and Leu237, it is observed that both exhibit less negative energy at 310 K, implying a reduction in hydrophobic stabilization at higher temperatures. For Ile289 and Ala290, these residues show relatively large changes, with Ala290 decreasing from −2.04 kcal/mol to −1.51 kcal/mol, suggesting a weakened hydrophobic interaction.

Interestingly, the Arg256 residue shows an increase in negative contribution from −0.50 kcal/mol at 298 K to −0.81 kcal/mol at 310 K. This could indicate a stabilizing effect at a higher temperature, possibly due to altered electrostatic interactions.

Val240 and Ser286 appear to have a measurable contribution at 298 K (−0.44 kcal/mol) but is absent at 310 K. Conversely, Ser286 contributes at 310 K (−0.21 kcal/mol) but not at 298 K. This suggests a shift in interaction patterns with temperature.

These results suggest that the ligand binding is slightly weakened at 310 K compared to 298 K, with specific residues such as Trp213, Arg217, and Ala290 showing significant reductions in their contributions. These changes reflect temperature-dependent shifts in hydrophobic and electrostatic interactions, which could impact the overall stability and affinity of the complex.

### 2.8. Temperature- and Ligand-Dependent Changes in Interaction Networks

To further analyze the conformational changes in BSA upon ligand association at 298 K and 310 K, the Residue Interaction Network Generator (RING) web service was employed. RING enables the identification of atomic interactions within a protein, allowing for a precise and quantitative analysis of conformation-dependent contacts [38,39].

**Figure 8:**
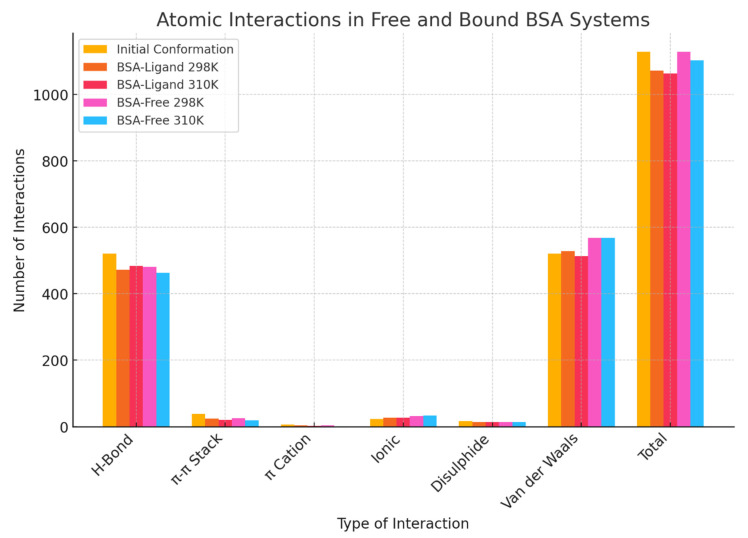
Atomic interactions in free and bound BSA systems. The bars represent the number of interactions observed in different systems, including the initial conformation, BSA–ligand at 298 K and 310 K, and free BSA at 298 K and 310 K. Interaction types include hydrogen bonds (H-Bond), π-π stacking, π-cation interactions, ionic interactions, disulfide bonds, and van der Waals interactions.

The most populated cluster structures from simulations at 298 K and 310 K, in both bound and free states, were compared with each other and with the initial conformation. For the MD simulations at both temperatures, the structures maintained a relatively constant number of atomic interactions (Figure 8). However, the presence of the ligand (bound form) appeared to reduce the total number of interactions at both temperatures, likely due to direct binding within the pocket. This observation also aligns with the RMSF observation of the free forms being more rigid.

Examining the specific types of interactions, the simulations resulted in the loss of π-π stacking, π-cation, and disulfide bonds, while increasing ionic interactions. Most of these trends followed a pattern where interactions were generally higher at 298 K or in the free form. In contrast, hydrogen bonds and van der Waals interactions did not exhibit a strong temperature- or ligand-dependent trend.

In addition, MD simulations enhanced van der Waals interactions while reducing the number of hydrogen bonds. For hydrogen bonding, the bound form at 298 K displayed fewer hydrogen bonds than its free counterpart, whereas at 310 K, the bound form exhibited a higher number of hydrogen bonds than the free form. Additionally, the free forms at both temperatures had a greater number of van der Waals interactions, while the bound form at 310 K had the lowest number of van der Waals interactions. This suggests that at 310 K, the presence of the ligand may promote hydrogen bond formation at the expense of van der Waals interactions, which may reflect higher conformation stability.

**Figure 9:**
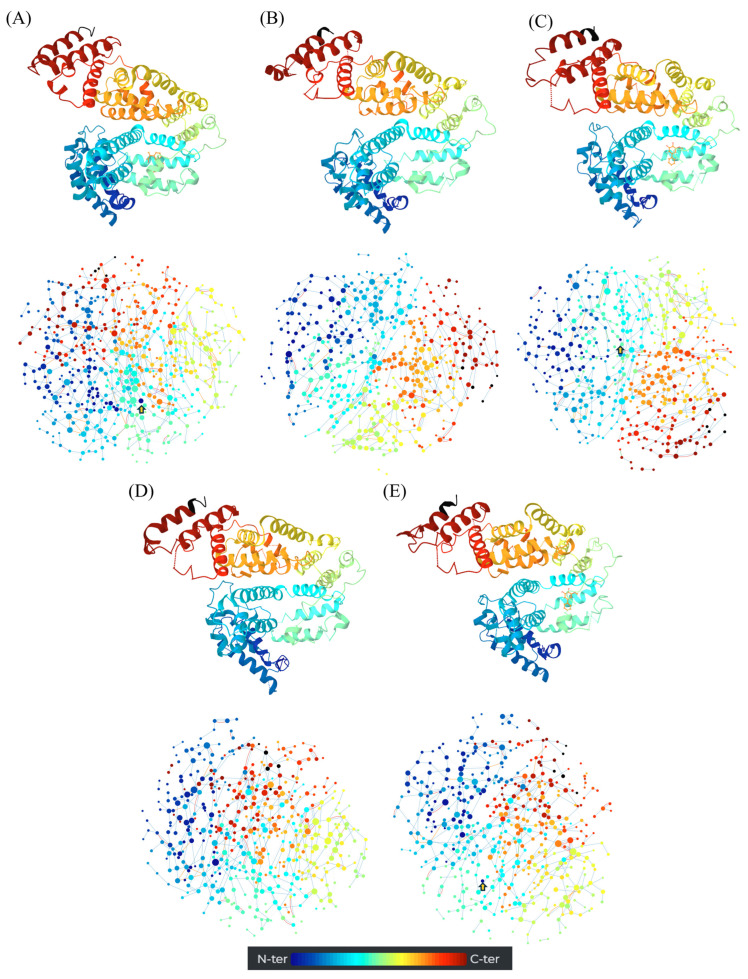
Graphical representation of BSA internal interactions: BSA interactions in both 3D structures (**top**) and RING network representation (**bottom**) of (**A**) BSA/ligand complex in its initial conformation, BSA at 298 K in (**B**) the apo and (**C**) the holo form, and at 310 K, in (**D**) the apo form and (**E**) the holo form. Coloring reflects the N- and C-termini. A yellow arrow indicates the ligand’s location within the RING network.

Furthermore, Figure 9 highlights significant alterations in conformation-dependent contacts, indicating temperature- and ligand-specific changes in the BSA’s interaction network. Overall, temperature appears to have the greatest influence on the network of residue interactions. Both forms of BSA at 298 K and 310 K are more similar to each other than to their respective counterparts at the other temperature (Figure 9A–E).

One of the most noticeable changes observed was the increased distance between the N- and C-terminal ends of the protein. At 298 K, in both the free and bound ligand forms, these ends appeared more separated compared to the initial structure and the systems at 310 K (Figure 9B,C vs. Figure 9A,D,E). This separation seems to be mediated by a nearby region close to the ligand’s binding site.

Additionally, at 298 K, the interaction network within the protein appeared more fragmented, with clearly separated colored regions (Figure 9B,C). This suggests a loss of long-range interactions between different parts of the protein. In contrast, the initial structure and the systems at 310 K (Figure 9A,D,E) showed a smoother color transition, indicating a more connected interaction network. Still, even in the ligand-bound form at 310 K, the termini showed some separation, though less pronounced than at 298 K.

Overall, both structures at 310 K resemble the initial conformation more closely. This observation contrasts with the total number of interactions, where both forms at 298 K exhibit a count more similar to the initial conformation than to the 310 K structures. Thus, this analysis suggests that temperature has a greater influence on the overall conformation, leading to distinct structural changes at each temperature.

**Figure 10:**
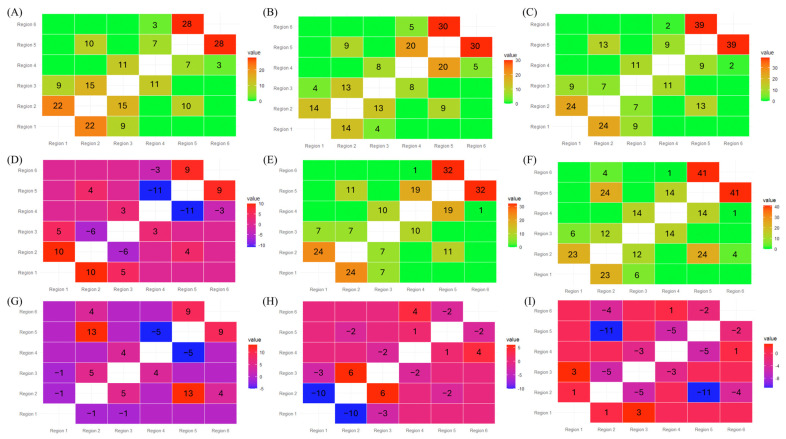
Heat map of inter-regional interactions: The BSA/ligand complex is shown at (**A**) its initial conformation, (**B**) 298 K, and (**C**) 310 K. (**D**) Interaction differences in the complex between 310 K and 298 K, with blue indicating enrichment at 298 K and red at 310 K. The BSA apo conformation is presented at (**E**) 298 K and (**F**) 310 K. (**G**) Interaction differences in apo BSA between 310 K and 298 K, following the same comparison color scheme. Differences between the BSA holo and apo states are shown at (**H**) 298 K and (**I**) 310 K, with red indicating an enrichment generated by the ligand.

To further analyze the data obtained from RING, the BSA structure was divided into distinct regions based on the distribution of α-helices. This segmentation allowed for a more systematic and focused analysis of structural dynamics and inter-regional interactions, allowing for the detection of differences within the structure and possible ligand-induced conformational changes. Specifically, the focus was on hydrogen bonds and the van der Waals interactions between different regions of the protein, which are represented in the heat map shown in Figure 10.

In general, MD simulations with the ligand maintain similar inter-regional interactions in both forms at 298 K (Figure 10B,E) and 310 K (Figure 10C,F) compared to the initial structure (Figure 10A). However, the number of interactions varies within each condition. In the initial structure, the first region interacts with both the second and third regions, with the interaction between regions 1 and 2 showing the second-highest number of interactions. Additionally, region 2 interacts with both regions 3 and 5. The interaction between regions 2 and 5 may explain the connection between the C- and N-termini observed in the RING networks.

Furthermore, region 3 interacts with region 4, region 4 with regions 5 and 6, and region 5 with region 6. Among these, the interaction between regions 5 and 6 has the highest number of inter-regional contacts. This suggests that the connectivity between the first and last thirds of BSA is primarily driven by interactions between regions 2 and 5. Meanwhile, interactions between the middle and other sections of the structure occur through the interaction of region 1 with region 3 and region 4 with region 6.

To quantify the conformational changes in BSA at 298 K and 310 K, the interaction differences between the two temperatures were calculated by subtraction, with blue indicating enrichment at 298 K and red indicating enrichment at 310 K (Figure 10D). In the bound form, 310 K enhances interactions between regions 1 and 3, regions 1 and 5, regions 2 and 5, regions 3 and 4, and regions 5 and 6. Conversely, at 298 K, the bound form favors interactions between regions 2 and 3, as well as among 4, 5, and 6.

For the free form (Figure 10G), the enhanced interactions among regions 1, 2, and 3 seen in the bound form at 310 K are no longer present. In contrast, the free form at 298 K shows slightly stronger interactions in these regions. Notably, the interaction between regions 2 and 3, which is favored at 298 K in the bound form, becomes more pronounced at 310 K in the free form. Additionally, the interaction between regions 2 and 5 is significantly enhanced in the free form at 310 K, along with a newly formed interaction between regions 2 and 6. These changes suggest that at 310 K, the structure favors interactions involving region 2 and region 5, contributing to a more interconnected network and a reduced distance between the C- and N-termini.

Furthermore, 310 K also enhances interactions between regions 3 and 4, as well as regions 5 and 6. In contrast, at 298 K, interactions between regions 4 and 5 are strengthened, similar to what is observed in the bound form. Overall, 310 K promotes interactions across distinct regions of BSA, with the most significant effects near the C- and N-termini (regions 2 and 5). Even though interactions appear stronger at 298 K, this analysis revealed that the interactions tend to be more localized within specific regions, rather than between them. This explains the compartmentalized color patterns observed in the RING networks at 298 K, while at 310 K, the interaction network appears more interconnected, particularly due to the enhanced interactions between regions 2 and 5. Finally, the comparison between free and bound forms highlights the ligand’s role in modulating specific interactions.

To further analyze the ligand’s contribution at both temperatures, interaction differences between the free and bound forms were calculated by subtraction, with red indicating ligands stimulated by the ligand (Figure 10H,I). Overall, the presence of the ligand reduces the number of interactions across most regions, as suggested by the total interaction count and the distribution within the RING network.

The ligand is consistently located in region 3 in both bound forms at 298 K and 310 K, yet it induces distinct rearrangements at each temperature.

At 298 K, the ligand disrupts interactions between region 3 and regions 1 and 4 while enhancing interactions with region 2. In contrast, at 310 K, the ligand primarily reduces interactions between region 3 and regions 2 and 4 while strengthening interactions with region 1. This indicates that, in both cases, the ligand destabilizes interactions involving region 3, but with distinct patterns: at 298 K, it promotes interactions with region 2, whereas at 310 K, it favors interactions with region 1. In both cases, interactions with region 4 are lost

At 298 K, the ligand causes a significant loss of interactions within region 1, particularly those connecting it to regions 2 and 3. This suggests that the ligand may increase the flexibility of region 1 at 298 K.

Furthermore, at both temperatures, the ligand disrupts interactions between regions 2 and 5, with this effect being particularly pronounced at 310 K. Notably, the free form at 310 K exhibits strong interactions between regions 2 and 5 and even between regions 2 and 6, but these interactions are lost in the bound form. However, the bound form still maintains four more interactions than the bound form at 298 K.

The ligand also affects region 5, reducing its interactions with region 6 at both temperatures. Interestingly, at 298 K, it enhances interactions between regions 6 and 4, whereas at 310 K, it weakens interactions between regions 4 and 5. In this way, the ligand induces structural rearrangements, primarily by disrupting inter-regional interactions.

The most striking effect at 298 K is the loss of interactions between region 1 and regions 2 and 3, likely contributing to the increased flexibility of region 1. At 310 K, the most significant change is the loss of interactions between regions 2 and 5, which contributes to the more compartmentalized network observed in the free form at this temperature compared to the free state. Considering conformational freedom based on inter-region interactions, the results suggest the following trend: bound at 298 K > free at 298 K > bound at 310 K > free at 310 K. To further examine this within the 3D structure, potential binding pockets were predicted using PrankWeb, selecting those with a probability higher than 0.70. The bound state at 298 K exhibited six pockets, the bound state at 310 K had four, and both free forms had five. These findings suggest that the binding of Noraucuparin at 298 K and 310 K has a different effect on the pocket availability within serum albumins.

## 3. Discussion

In contrast to the experimental and docking-based findings previously reported by Dai et al. (2025) [26], our study employs long-timescale MD simulations—three independent 1-μs replicas per system—combined with MMGBSA analyses to provide a deeper understanding of the dynamic and energetic features of the BSA–noraucuparin complex. This extended simulation approach revealed that ligand binding not only stabilizes the complex but also enhances the conformational flexibility of BSA, particularly at 298 K. These temperature- and ligand-dependent changes, including the expansion of the conformational space, restructuring of intramolecular interaction networks, and modulation of binding pocket dynamics, are insights that would remain inaccessible through static or short-timescale methodologies. Our results, therefore, extend and refine previous observations by revealing how noraucuparin binding modulates the global dynamics of BSA in a temperature-sensitive manner.

Our MD simulations of the BSA–noraucuparin complex revealed that ligand binding not only stabilizes the protein but also increases its conformational flexibility, particularly at 298 K. This aligns with previous studies demonstrating ligand-induced flexibility in BSA, such as the work by Morrisett et al. (1975) [40], who showed that fatty acid binding increases protein mobility in a temperature-dependent manner. Their findings identified two temperature-driven conformational transitions (15–23 °C and 38–45 °C), similar to our observation that ligand-induced flexibility is more pronounced at 298 K than 310 K.

This phenomenon, where a ligand increases rather than restricts protein mobility, has been observed in other receptor–ligand systems and can be attributed to several structural and energetic factors, including allosteric effects, entropy-driven binding, and dynamic equilibrium shifts within the protein’s energy landscape. This observation is consistent with previous studies documenting cases where ligand binding enhances protein mobility, rather than restricting it.

The scientific literature supports the notion that ligand binding can increase the receptor mobility, as observed in various biological systems. For instance, studies on the β_2_-adrenergic receptor (β_2_AR), a well-characterized GPCR, have demonstrated that ligand binding can enhance receptor flexibility. Research combining nuclear magnetic resonance (NMR) spectroscopy with MD simulations revealed that upon agonist binding, the extracellular region of β_2_AR becomes more stabilized, while the intracellular region exhibits increased conformational variability [41]. Another example involves the lactose repressor protein (LacI), where ligand-specific changes in conformational flexibility mediate long-range allosteric regulation. Binding of different ligands to LacI results in distinct alterations in the protein’s rigidity and conformational ensemble, influencing its regulatory functions [42]. These studies suggest that increased flexibility upon ligand binding is a broader biological principle, rather than an exception, and may serve functional roles in molecular recognition and allosteric regulation.

In the case of the BSA–noraucuparin complex, MD simulations revealed that ligand binding alters the protein’s conformational landscape, in line with previous studies that showed that noraucuparin binding induces structural modifications in BSA [26], increasing its mobility, especially at 298 K. Notably, per-residue free energy decomposition identified key residues, such as Trp213, Arg217, and Leu237, as crucial for the binding stability. These residues undergo conformational rearrangements upon ligand association, contributing to the increased structural flexibility of BSA. The presence of noraucuparin disrupted specific intramolecular interactions within BSA, resulting in an expanded conformational phase space.

Although ITC suggests an increase in binding affinity at higher temperatures [26], while MMGBSA indicates overall stability with only minor variations between 298 K and 310 K, the reduced conformational flexibility of the BSA–noraucuparin complex at 310 K may contribute favorably to binding by minimizing the entropic penalty (-TΔS). This reduction in mobility could help stabilize the complex, making binding more thermodynamically favorable at higher temperatures.

Similarly, studies examining BSA at 303 K in aqueous and reline solutions reported increased RMSF values at 50% reline concentration, attributed to a closer approach of domains II and III and a concurrent loss of interactions between domains I and III [43]. In our analysis, these domains correspond to regions 1 and 2 (domain I), regions 3 and 4 (domain II), and regions 5 and 6 (domain III). Our inter-regional interaction data show that at 298 K, both the apo and holo forms exhibit enhanced interactions between regions 4 and 5, while ligand binding further strengthens contacts among regions 4, 5, and 6 compared to 310 K (Figure 10). The observed separation between domains I and III mirrors the rearrangements at the N- and C-termini (Figure 9 and Figure 10). In contrast, the conformational organization at 310 K resembles that of BSA in aqueous solution, where domains I and II or III are more tightly packed.

This difference is particularly relevant given the conformational rearrangements observed at 298 K in both pure water (Figure 9 and Figure 10) and reline–water mixtures near 303 K [43], where BSA displays increased flexibility and likely a looser hydration shell. Hydration shells are known to play a crucial role in the thermodynamics of binding and in the accuracy of computational predictions. Indeed, studies have shown that explicitly including hydration shells in MMGBSA calculations significantly improves the correlation between computed binding affinities and experimental data [44,45].

Therefore, at 298 K, ligand binding is associated with increased protein flexibility and a less structured hydration environment, leading to a higher conformational and solvent entropy that favorably contributes to the overall binding process. At 310 K, in contrast, the complex adopts a more rigid conformation with tighter domain packing and a more ordered hydration shell. These opposing behaviors suggest that hydration and entropy changes are key contributors to the differences observed between the two temperatures—effects that are captured by ITC but typically underestimated by MMGBSA.

This interpretation is consistent with findings from other protein–ligand systems where temperature-dependent shifts in the energy landscape influence both binding affinity and conformational dynamics. Even within BSA, different thermodynamic signatures have been reported. For example, Cd^2+^ binding is largely enthalpy-driven, with unfavorable entropy contributions reflecting reduced flexibility and increased solvation order [46], whereas Congo red binding involves both enthalpic and entropic components associated with conformational changes and water reorganization [47].

In summary, the similar ΔGbind values at 298 K and 310 K likely result from a compensation between enthalpic and entropic effects. While higher temperatures promote more favorable hydrogen bonding and domain packing (enthalpic contributions), lower temperatures enhance flexibility and solvent disorder (entropic contributions), ultimately leading to comparable overall binding-free energies.

One particularly intriguing finding was that ligand-induced flexibility was more pronounced at 298 K than at 310 K. This suggests that the temperature modulates ligand-induced dynamics, with lower temperatures favoring a more extensive conformational exploration. This observation is consistent with studies on other protein–ligand systems, where temperature-dependent energetic landscapes influence the binding affinity and structural rearrangements. Moreover, interaction network analyses using RING demonstrated that the number of total interactions decreased upon ligand binding, reinforcing the idea that ligand association destabilizes specific intramolecular contacts, leading to a more dynamic protein structure.

The observation that ligand binding enhances conformational mobility has important implications for pharmacokinetics and drug delivery. Increased flexibility may improve the ability of serum albumins to accommodate various ligands, influencing their transport and distribution in the bloodstream. Additionally, the balance between stabilizing interactions and dynamic fluctuations could be a key factor in determining the ligand affinity and specificity. Future experimental validation using techniques, such as fluorescence anisotropy, circular dichroism, and hydrogen-deuterium exchange mass spectrometry (HDX-MS), could provide further insights into the dynamic behavior of the BSA–ligand complex. A comparative analysis with other biphenyl-type ligands may also reveal general principles governing ligand-induced conformational mobility in serum albumins.

## 4. Materials and Methods

### 4.1. Molecular Docking

The structure of BSA was obtained from the Protein Data Bank (PDB entry 6QS9), where BSA forms a complex with Ketoprofen. The missing loops in BSA were reconstructed using SWISS-MODEL [48]. The stereochemical quality of the protein model was evaluated using a Ramachandran plot generated with RamPlot. A total of 96.0% of residues were located in favored regions, 3.4% in allowed regions, and only 0.6% in disallowed regions [49]. The structure of the Noraucuparin molecule was downloaded from the ChemSpider database (ChemSpider: Search and Share Chemistry—Homepage) and optimized using Avogadro [50], combining the UFF force field with the steepest descent approach, further refined using conjugate gradient algorithms. Docking experiments were performed with SwissDock [51], applying blind docking to define the search area and using standard settings. The receptor–ligand configuration with the lowest binding energy was selected as the representative complex. PyMOL [52] was used to visualize the results from SwissDock. The docking method was validated by redocking Ketoprofen onto BSA, yielding an RMSD below 1.0 Å (see Supplementary Figure S5).

### 4.2. MD Simulations

All MD simulations were performed using the Amber22 package. The protein–ligand complexes were solvated in a truncated octahedral box filled with TIP3P water molecules, ensuring a minimum distance of 10 Å between the solute and the box boundary. Appropriate counterions (Na^+^ or Cl^−^) were added to neutralize the systems. The following procedures were used to minimize and equilibrate the systems prior to the MD simulations. Four thousand iterations of conjugate gradient minimization and five thousand iterations of steepest descent were used in the minimizing process. Using a Berendsen thermostat [53], the systems’ temperature was increased by 200 ps from 0 to 298 or 310 K while keeping an NVT ensemble. The protein’s heavy atoms were constrained during this process by an elastic constant of 3 kcal mol^−1^ Å^−2^. A Berendsen barostat [53] and a Langevin thermostat [54,55] were then used to equilibrate the density for 200 ps in an NTP ensemble. A constant pressure was maintained for 600 ps to achieve equilibration at 298 K or 310 K, while maintaining the same constraints on the heavy atoms that were applied during the heating phase. MD simulations were conducted as described above, but using an NTP ensemble without imposing any constraints. The particle mesh Ewald (PME) [56] approach was used to address the long-range electrostatic interactions, whereas the van der Waals forces and short-range electrostatic interactions were determined with a cutoff of 10 Å. The SHAKE algorithm [57] was used to maintain the link lengths between hydrogen atoms and their related heavy atoms during the simulations, which used a time step of 2 fs. The pmemd.cuda module in Amber22 [58] was used to perform the MD simulations in triplicate. A unified trajectory was generated for each system by concatenating the three simulations of one microsecond each, from which the RMSD, RMSF, Rg, clustering analysis, and ΔGbind calculations were determined. Figures were generated using PyMol [52] software.

### 4.3. Structural Interaction Network and Regional Analysis

To evaluate how ligand binding affects residue-residue interactions within the protein at 298 K and 310 K, we used RING v4 to map non-covalent atomic-level interactions across the structure [39]. The minimized and equilibrated structure of the BSA–ligand complex was used as the initial conformation, while the most populated cluster structures from simulations at 298 K and 310 K served as representative conformations at each temperature in both apo and holo forms. Moreover, the RING web service was used with default settings, including closest nodes, multiple edges, a strict threshold, and default Ångström cutoffs for different types of interactions. The graphical representation of the network and structure, along with the contact table information, was obtained directly from the RING webpage. The division of BSA was based on the number of α-helices present in its structure. In the initial conformation, 25 α-helices were identified, which were grouped into six main regions for analysis. These regions were defined as follows: Region 1: Lys4 to Lys93, Region 2: Phe96 to Gly206, Region 3: Glu207 to Val292, Region 4: Leu301 to Lys396, Region 5: Glu399 to Val481, and Region 6: Asn482 to Ala583. Finally, PrankWeb: Ligand Binding Site Prediction, with the Default prediction model, was used to evaluate the possible binding pockets within the structures [59].

### 4.4. Binding-Free Energy and Per-Residue Decomposition Calculations

The final 200 ns of the triplicate one-microsecond MD trajectories were used to compute the binding-free energy for each system. This was achieved using the MMGBSA approach and the single-method MD simulation protocol, implemented through the MMPBSA.py module [60] in the Amber22 simulation program [58]. As described in previous studies, these approaches allow the binding-free energy to be decomposed into its energetic components [61].

## 5. Conclusions

Through extensive MD simulations and MMGBSA calculations, this study provides a comprehensive characterization of the molecular interactions governing the binding of noraucuparin to BSA. Our results indicate that noraucuparin binds to site II of BSA with high stability, primarily through hydrophobic interactions and hydrogen bonding. Structural flexibility analyses demonstrated that ligand binding increases protein conformational mobility, particularly at 298 K, where a broader conformational landscape was observed. Energetic decomposition identified key residues involved in stabilizing the complex, with Arg217 playing a central role in hydrogen bonding, while Trp213 contributed via π-π interactions. The interaction network analysis further confirmed temperature-dependent rearrangements in intramolecular contacts, highlighting the ligand’s influence on BSA’s global dynamics.

Altogether, these findings not only deepen our understanding of albumin–ligand interactions but also underscore the potential of noraucuparin as a pharmacologically relevant compound with promising binding properties. The methodology used in this study may be extended to explore the interaction of other natural compounds with serum albumins, providing a valuable computational framework for early-stage drug development and carrier system design. Future studies integrating experimental validation, such as fluorescence spectroscopy or isothermal titration calorimetry, will be essential to corroborate these computational predictions and deepen our understanding of ligand-induced conformational changes in serum albumins.

## Data Availability

The datasets generated and analyzed during the current study are available from the corresponding authors upon request.

## Supplementary Materials

The following supporting information can be downloaded at https://www.mdpi.com/article/10.3390/ph18071048/s1, Figure S1: RMSD analysis of free and bound BSA at 298 and 310 K. (A) BSA_free-298K_, (B) BSA_free-310K_, (C) BSA_bound-298K_, and (D) BSA_bound-310K_. The black, blue, and red lines correspond to three different MD simulations for each system. Figure S2. Rg analysis of free and bound BSA at 298 and 310 K. (A) BSA_free-298K_, (B) BSA_free-310K_, (C) BSA_bound-298K_, and (D) BSA_bound-310K_. The black, blue, and red lines correspond to three different MD simulations for each system. The similarities in values across the three simulations make it difficult to observe any differences for BSA_bound-310K_. Figure S3. Surface area analysis of free and bound BSA at 298 and 310 K. (A) BSA_free-298K_, (B) BSA_free-310K_, (C) BSA_bound-298K_, and (D) BSA_bound-310K_. The black, blue, and red lines correspond to three different MD simulations for each system. Figure S4. Occupancy of the hydrogen bond between Arg217 and Noraucuparin at 298 K and 310 K. Figure S5. Superposition of the crystallized pose of ketoprofen (cyan) and the redocked pose (green) in the BSA binding site. The low RMSD (<1 Å) confirms the reliability of the docking protocol. Figure S6. Comparison of binding-free energy (ΔG) values for noraucuparin–BSA complexes at 298 K and 310 K across four time intervals (0.2–1.0 μs), calculated using MMGBSA from three replicate MD simulations.
