# Supplementary material for: Molecular Insights into the Temperature-Dependent Binding and Conformational Dynamics of Noraucuparin with Bovine Serum Albumin: A Microsecond-Scale MD Simulation Study
Source: Pharmaceuticals (Basel). 2025 Jul 17;18(7):1048. doi: 10.3390/ph18071048 (PMC12300129; doi:10.3390/ph18071048)
Supplement: Supplementary file 1 [file pharmaceuticals-18-01048-s001.zip › pharmaceuticals-3730129-supplementary.pdf]

Supplementary Material

Molecular Insights into the Temperature-Dependent Binding and Conformational Dynamics of Noraucuparin with Bovine Serum Albumin: A Microsecond-Scale MD Simulation Study

Erick Bahena-Culhuac [1,2] and Martiniano Bello [1,*]

[1] Laboratorio de Diseño y Desarrollo de Nuevos Fármacos e Innovación Biotecnológica, Sección de Estudios de Posgrado e Investigación, Escuela Superior de Medicina, Instituto Politécnico Nacional, Plan de San Luis y Salvador Diaz Mirón s/n, Casco de Santo Tomás, Miguel Hidalgo, Ciudad de México 11340, Mexico

[2] Faculty of Biology and Medicine, University of Lausanne, 1015 Lausanne, Switzerland

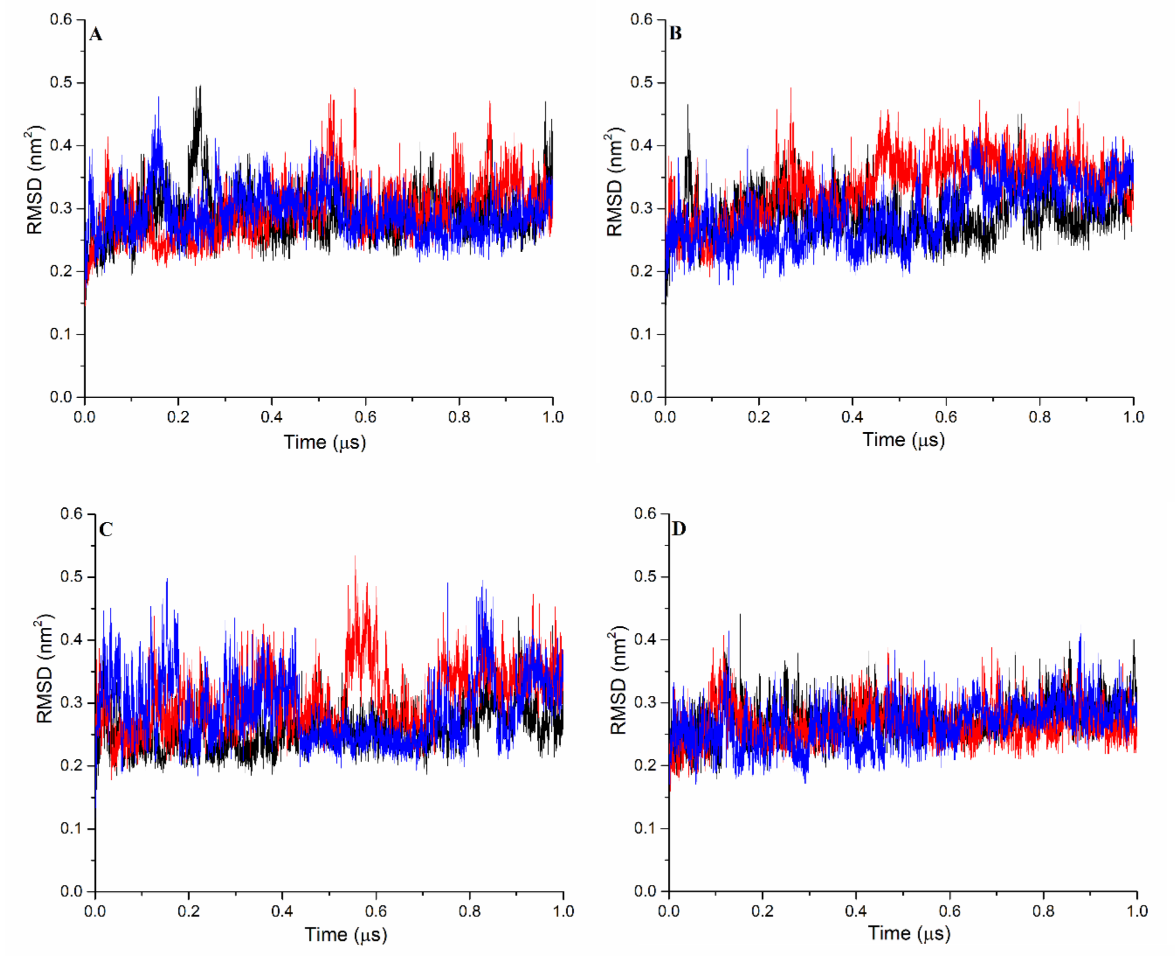

Figure S1. RMSD analysis of free and bound BSA at 298 and 310K. A) $BSA_{free-298K}$, B) $BSA_{free-310K}$, C) $BSA_{bound-298K}$, and D) $BSA_{bound-310K}$. The black, blue, and red lines correspond to three different MD simulations for each system.

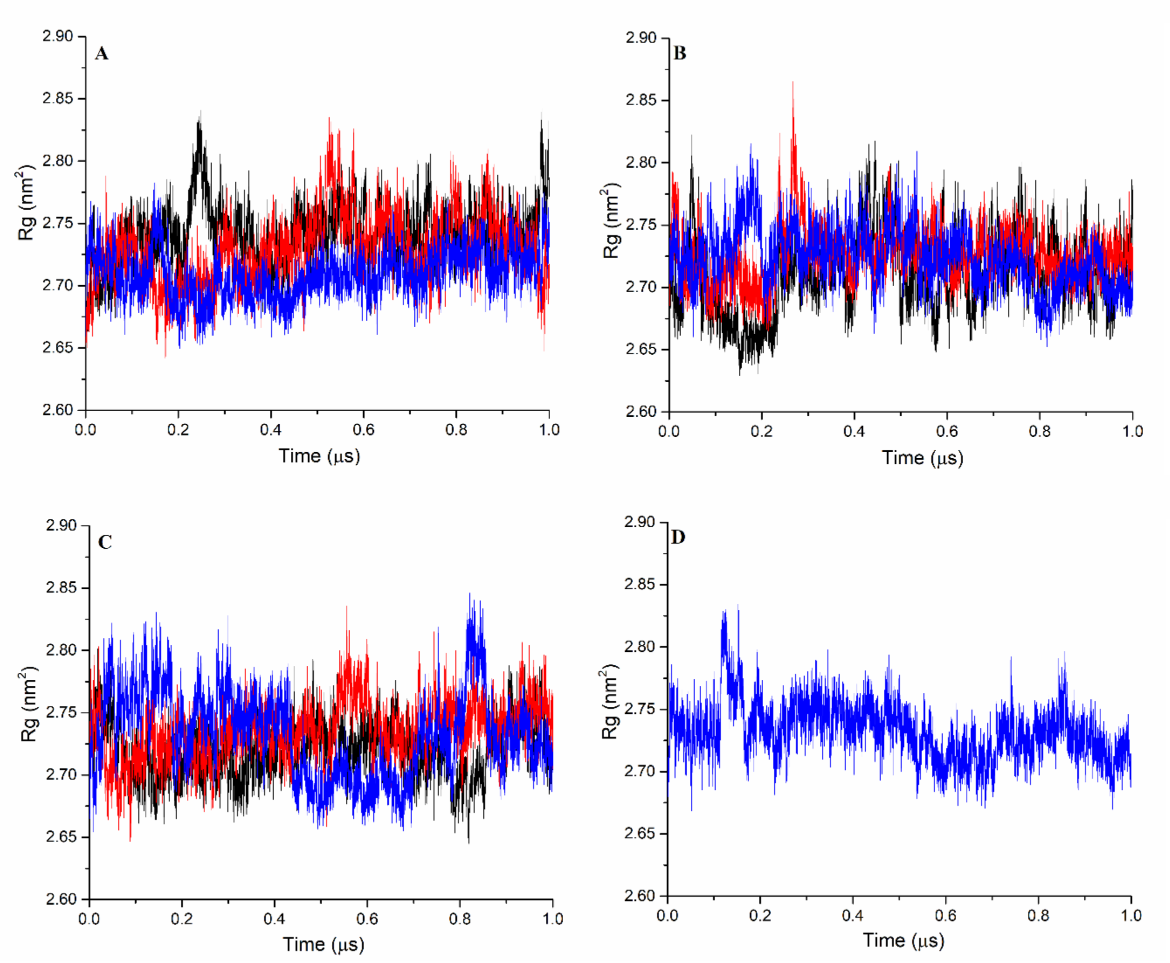

Figure S2. Rg analysis of free and bound BSA at 298 and 310K. A) $BSA_{free-298K}$, B) $BSA_{free-310K}$, C) $BSA_{bound-298K}$, and D) $BSA_{bound-310K}$. The black, blue, and red lines correspond to three different MD simulations for each system. The similarities in values across the three simulations make it difficult to observe any differences for $BSA_{bound-310K}$.

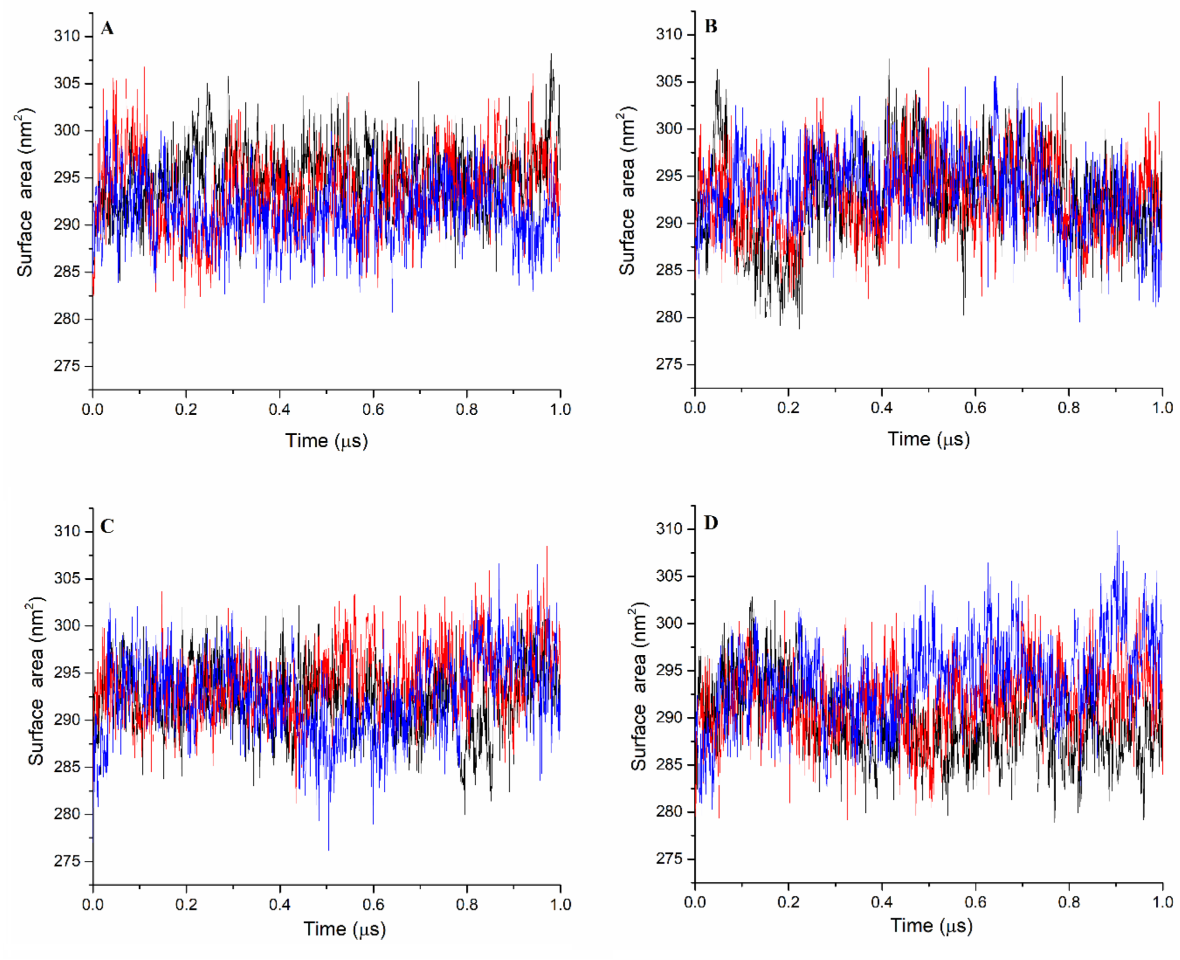

Figure S3. Surface area analysis of free and bound BSA at 298 and 310K. A) $BSA_{free\text{-}298K}$, B) $BSA_{free\text{-}310K}$, C) $BSA_{bound\text{-}298K}$, and D) $BSA_{bound\text{-}310K}$. The black, blue, and red lines correspond to three different MD simulations for each system.

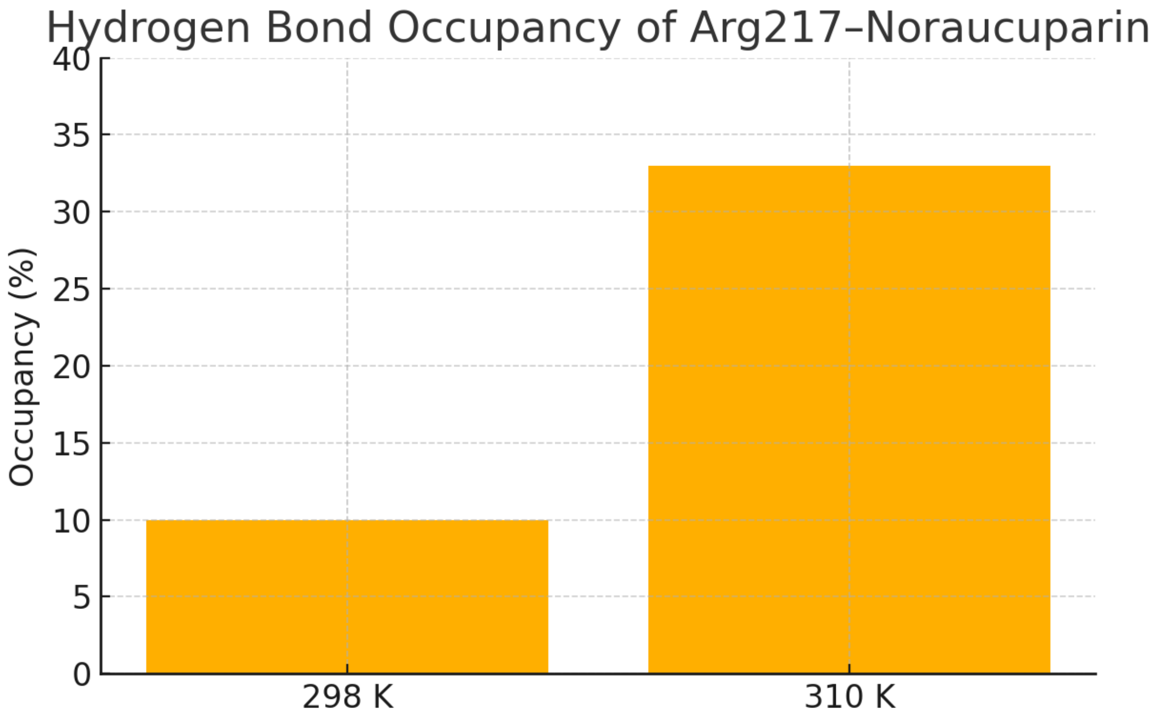

Figure S4. Occupancy of the hydrogen bond between Arg217 and Noraucuparin at 298 K and 310 K.

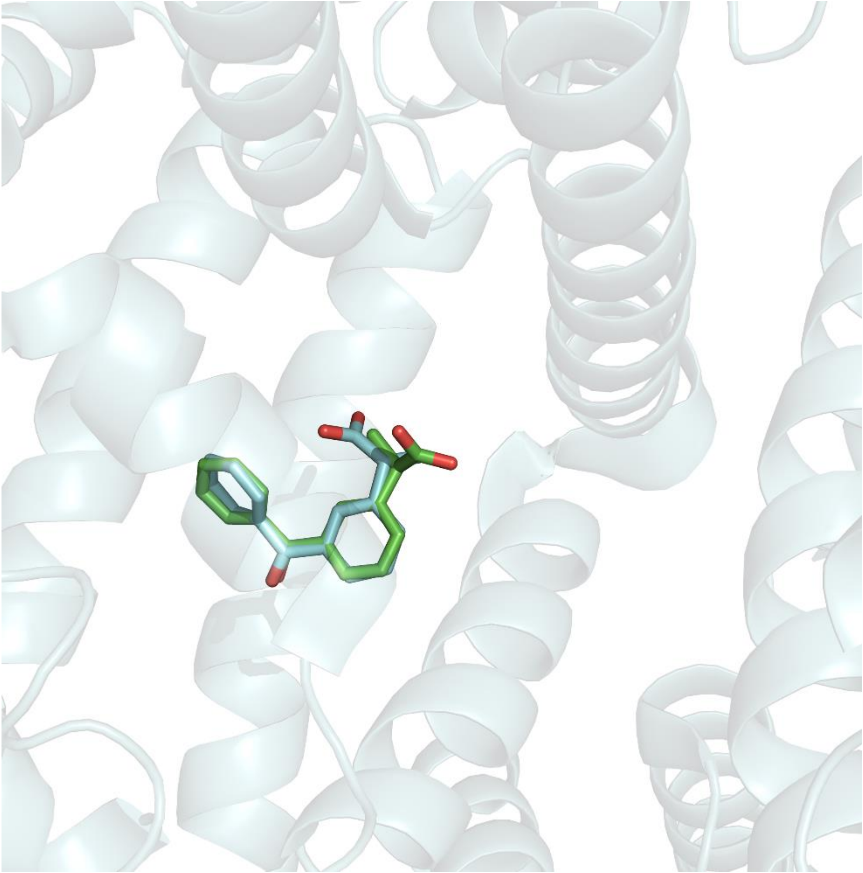

Figure S5. Superposition of the crystallized pose of ketoprofen (cyan) and the redocked pose (green) in the BSA binding site. The low RMSD (< 1 Å) confirms the reliability of the docking protocol.

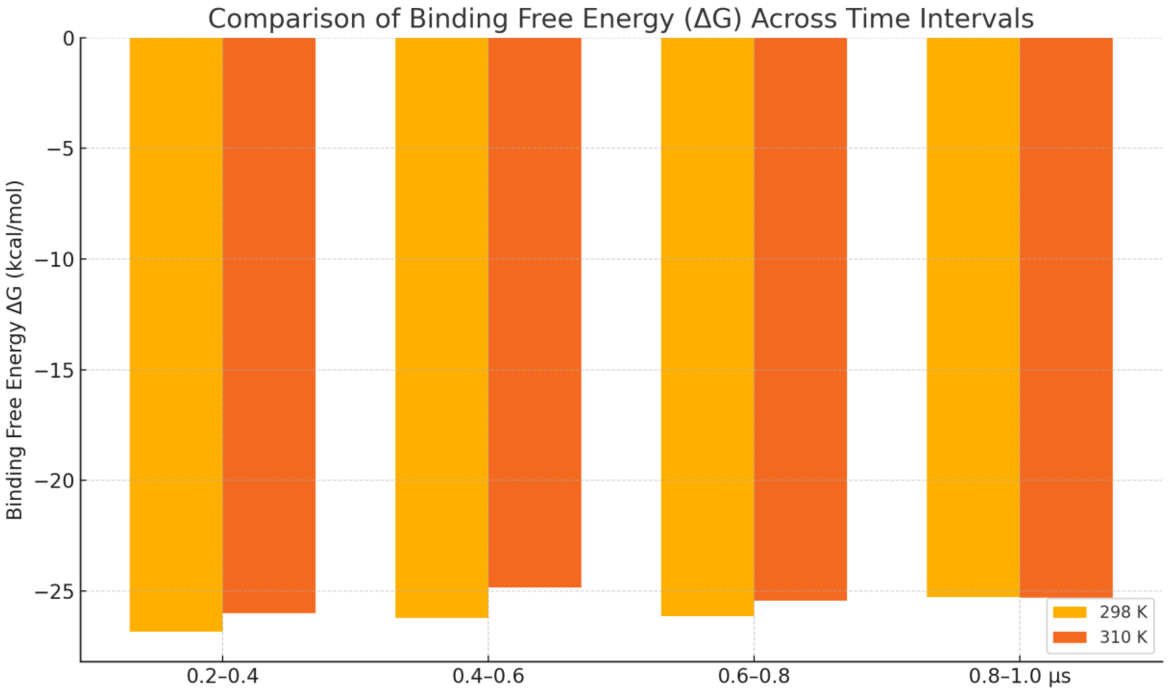

Figure S6. Comparison of binding free energy (ΔG) values for noraucuparin–BSA complexes at 298 K and 310 K across four time intervals (0.2–1.0 μs), calculated using MMGBSA from three replicate MD simulations.
